# Supplementary material for: Multi-proxy analyses of a mid-15th century Middle Iron Age Bantu-speaker palaeo-faecal specimen elucidates the configuration of the ‘ancestral’ sub-Saharan African intestinal microbiome
Source: Microbiome. 2020 May 6;8:62. doi: 10.1186/s40168-020-00832-x (PMC7204047; doi:10.1186/s40168-020-00832-x)
Supplement: Supplementary file 2 — Additional file 1: Figure S1. Additional information concerning the archaeological provenance of the BRS faecal specimen. Figure S2. Processing of the faecal specimen at the Centre for GeoGenetics, Copenhagen, Denmark. Figure S3. Dot-plot indicating the occurrence of statistically-significant C-T p-values. Figure S4. Biplot of δ13C and δ15N stable isotope values obtained for the BRS specimen. Figure S5. SEM analysis detected bacterial cells, plant fragments and saprophytic organisms. Figure S6. Heat-map indicating differences in taxonomic community structure for IM datasets. Figure S7. Comparing ‘relative abundance’ and ‘presence-absence’ as taxonomic representation. Figure S8. Comparison of the incidence of the twenty-four authenticated ancient IM taxa. Figure S9. Heat-map indicating the presence of fifteen functional ARGs identified. [file 40168_2020_832_MOESM1_ESM.docx]

**Supplementary materials**

**Supplementary Figures**

**
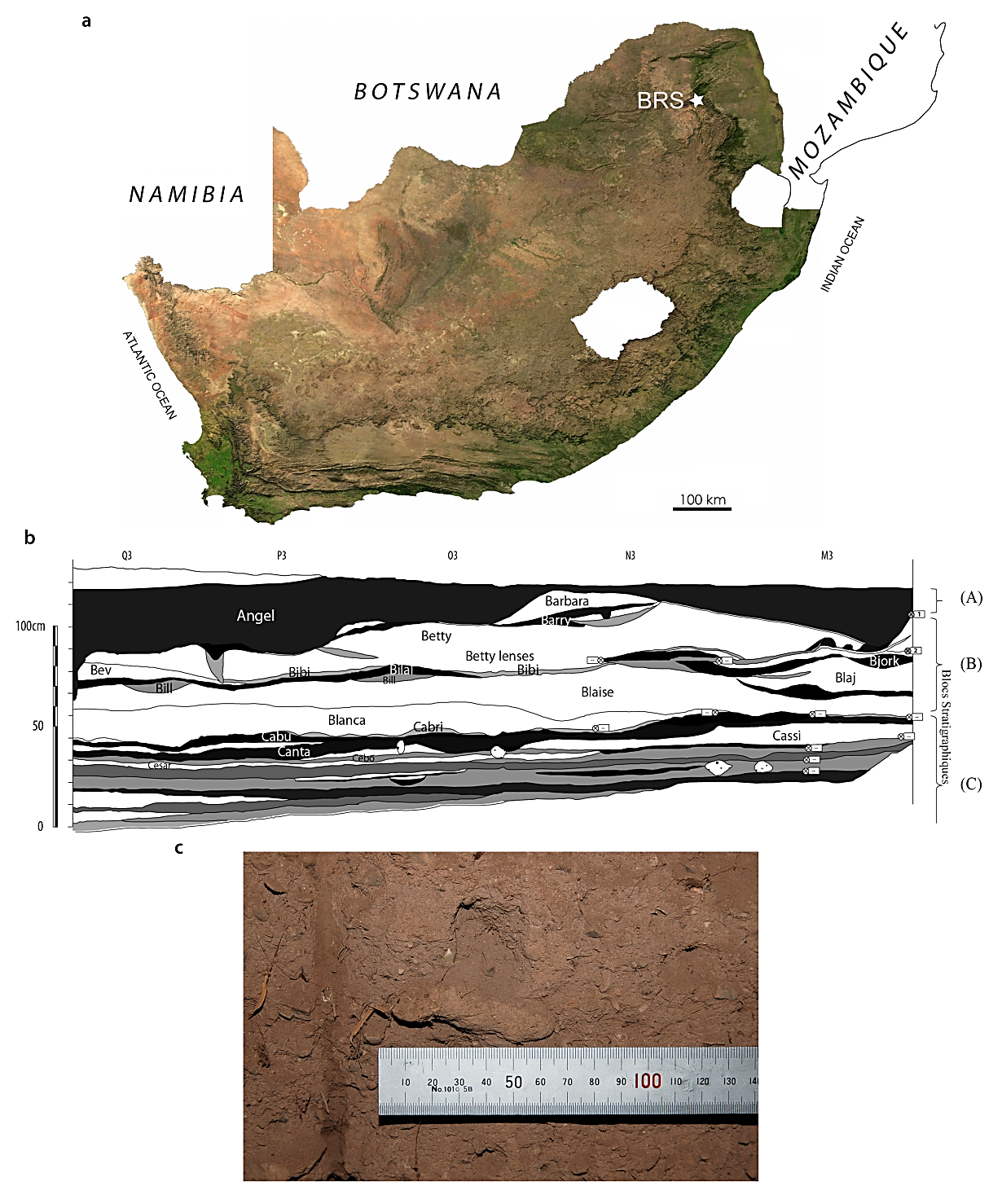
**

**Fig. S1.** The provenience of the BRS palaeo-faecal specimen. The location of Bushman Rock Shelter (BRS) in Limpopo Province, South Africa (**A**), stratigraphic proﬁle of the Iron Age occupation level from which the specimen derives and which comprise the upper layer of the rock-shelter (Layer 1) situated in excavation ‘Block A’ and designated ‘Angel’ (**B**) and the palaeo-faecal specimen *in situ* in the exposed (excavated) section prior to removal from the deposit (**C**).

**
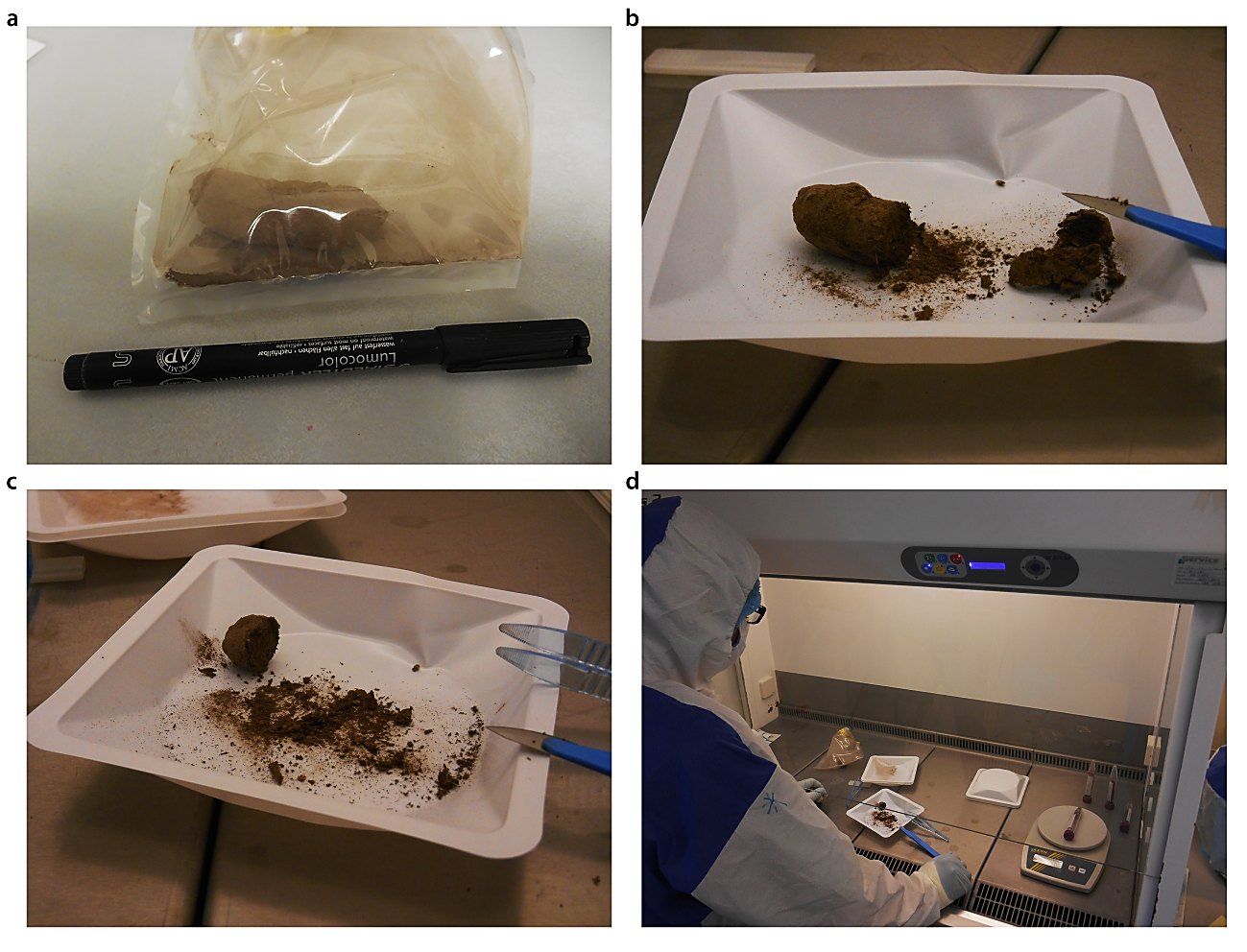
**

**Fig. S2.** Processing (sub-sampling and aDNA extraction) protocol applied to the BRS palaeo-faecal specimen. The frozen specimen was first removed from the sealed packaging (**A**), after which the outer surface or cortex (~5mm) was removed with a scalpel (**B**) and subsequently sub-sampled for radiocarbon (14C) dating and isotopic and microscopic intestinal parasitic analyses, preserving one-sixth of the specimen (at -20°C) as a voucher sample (**C**). Sub-sampling and aDNA extraction and library preparation was performed in the ‘clean’ ancient DNA laboratories at the Centre for GeoGenetics, University of Copenhagen (Denmark) (**D**).

**
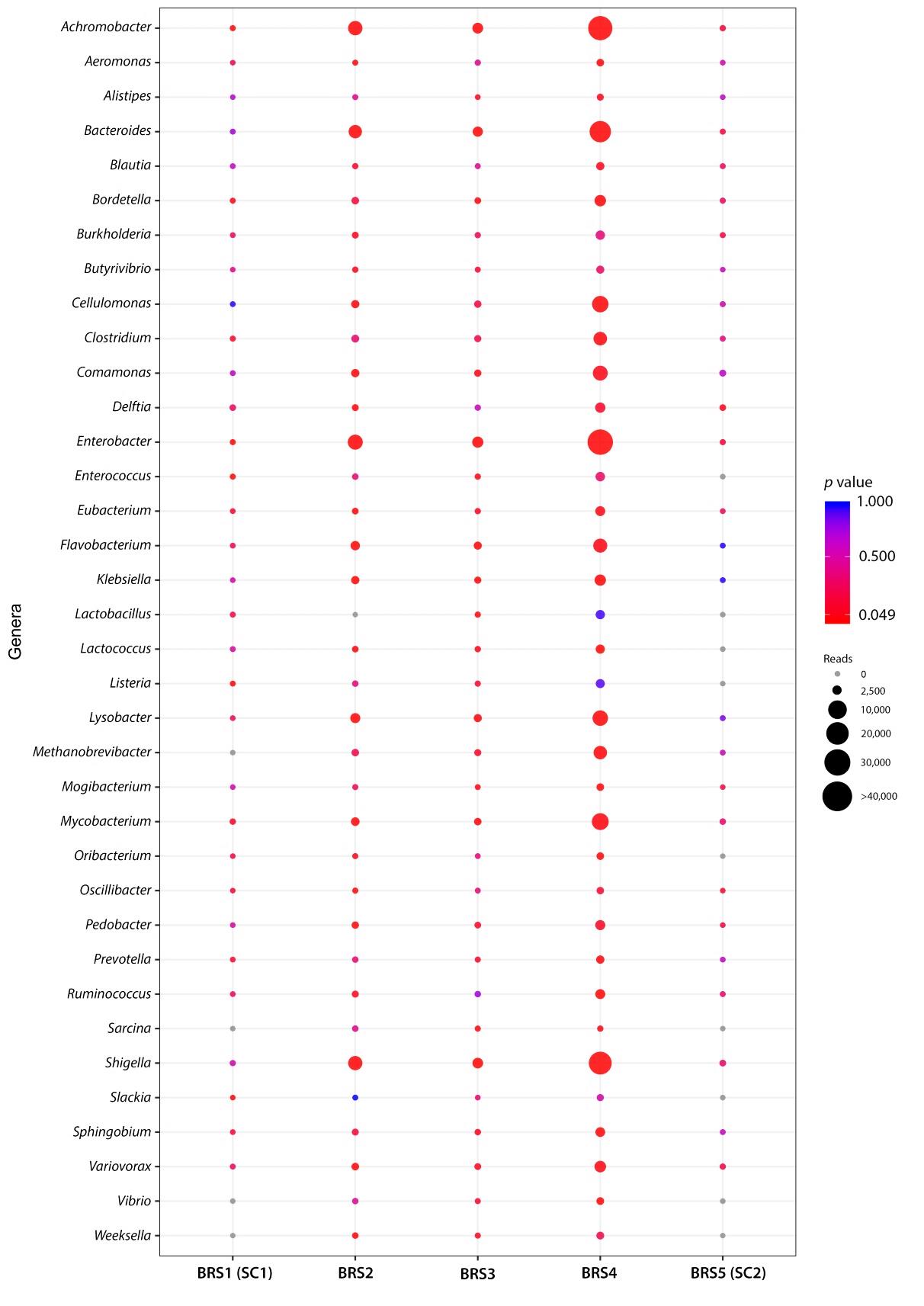
**

**Fig. S3.** Dot-plot based on the alignment of high-quality DNA sequence reads indicating the occurrence of statistically-significant C-T *p*-values calculated for commensal and pathogenic taxa detected in the BRS specimen (BRS2, BRS3 and BRS4) and the sedimentary controls (BRS1 or ‘SC1’ and BRS5 or ‘SC2’). Circle sizes and colours represent mapped read counts and *p*-value significance, respectively (see legend and scale).

**Fig. S4.** Biplot of δ^13^C and δ^15^N stable isotope ratios obtained for the BRS specimen. The various pre-treatments (*i.e.*, the acid wash and lipid extraction) had some effect on both δ^13^C and δ^15^N values. The lipid extraction (2:1 chloroform/ethanol) removed traces of lipids with carbon isotope values becoming less negative, but nevertheless suggesting a mixed C4- (mostly) and C3-based meal. The results for the solvent residue reflect a geological signal most likely from the shelter’s sediments. Average values for ‘untreated’, ‘lipid extracted’ and ‘acid washed’ are indicated in corresponding black markers.

**
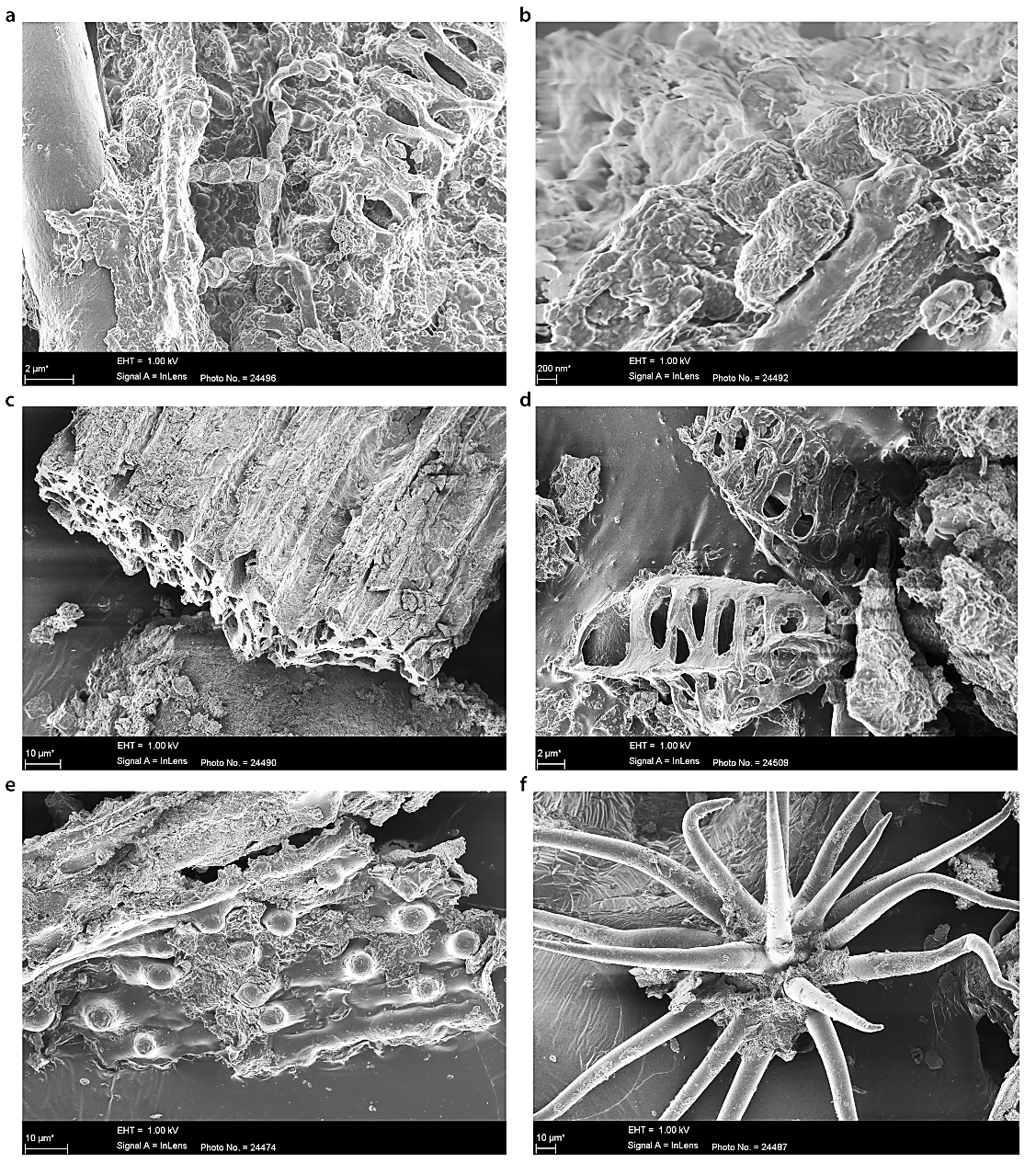
**

**Fig. S5.** Scanning electron microscopy (SEM) analyses resulted in the detection of desiccated bacterial cells (**A** and **B**), degraded plant fragments (**C**, **D** and **E**) and unidentified plant stellate hair (**F**).

**
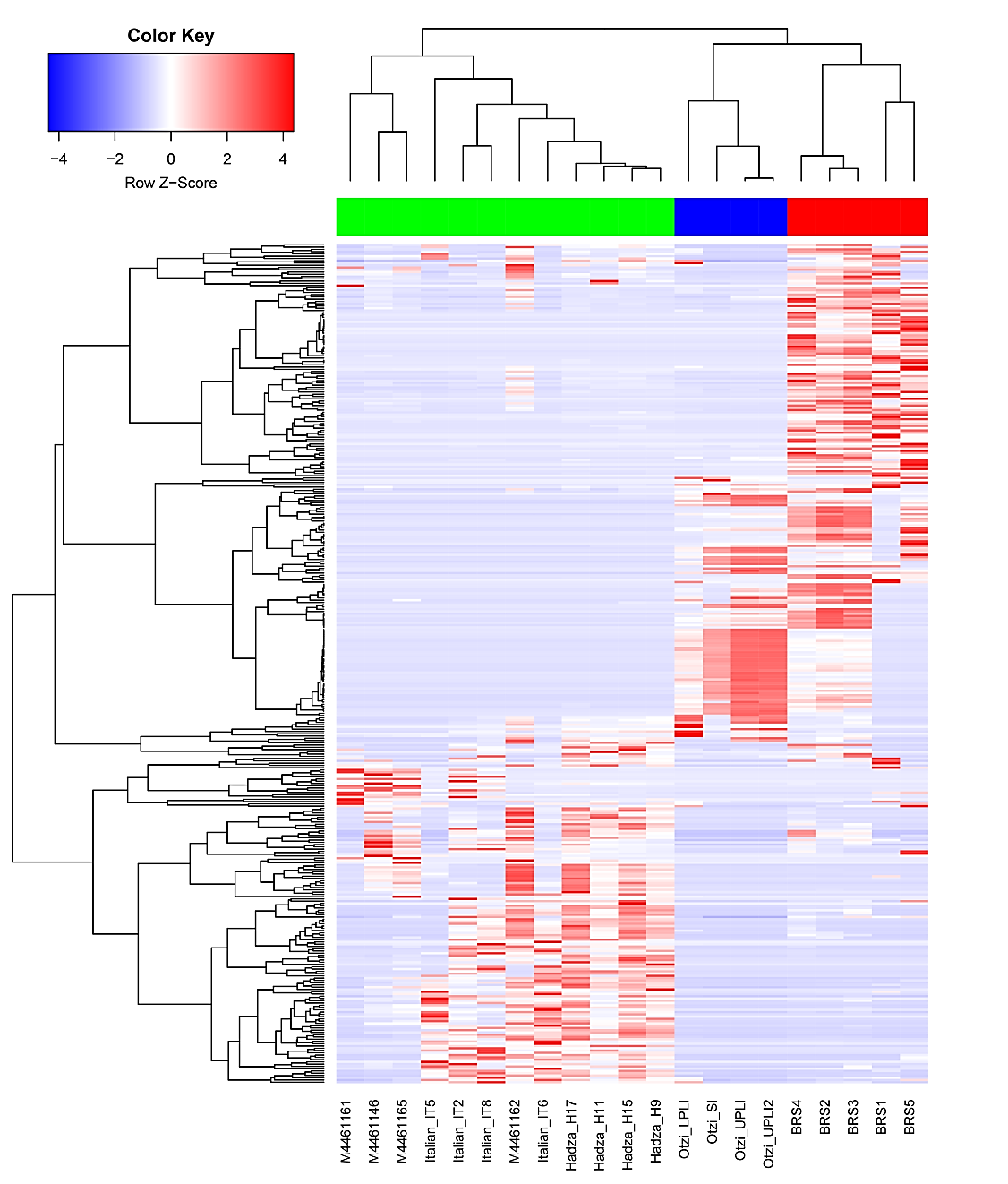
**

**Fig. S6.** Heat-map comparing differences in taxonomic community structure for BRS (1, 2, 3, 4 and 5) with the modern (Italian), ethnographic (Hadza and Malawian) and ancient (Ötzi) IM datasets. Hierarchical clustering using complete linkage based on Spearman's correlation, produced a clear separation between ancient (BRS and Ötzi) and modern (Italian, Hadza and Malawian) populations. ANOSIM analysis revealed significant differences between the ancient and modern IM samples (*R* ꞊ 0.6111; *p* ꞊ < 0.05) in the taxonomic categories of 731 taxa. Taxa were filtered for occurrence of >3 in at least 20% of the samples.

**
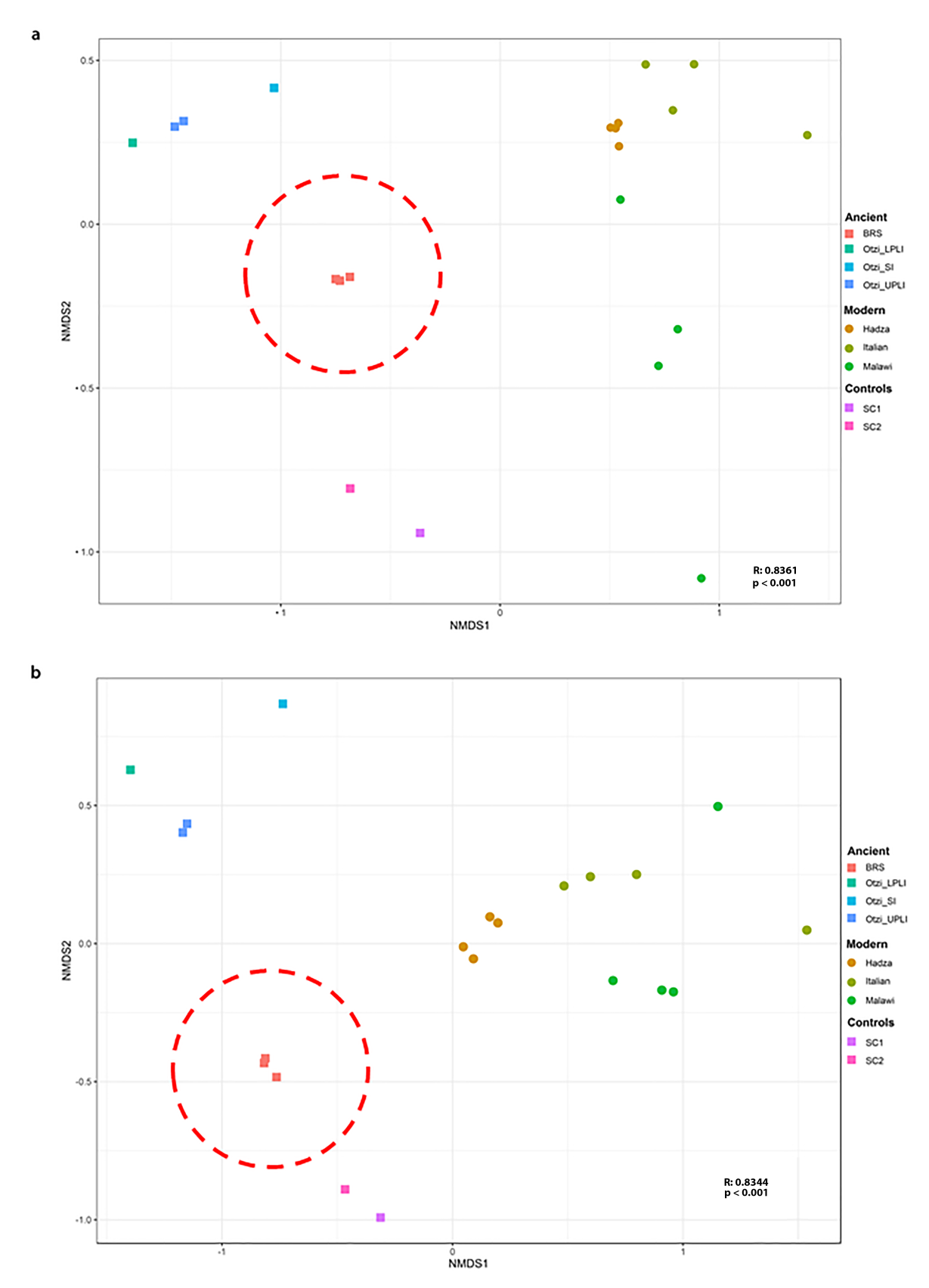
**

**Fig. S7.** Weighted (**A**) and un-weighted (**B**) Bray-Curtis non-metric multi-dimensional scaling (NMDS) plots comparing the use of ‘relative abundance’ (indicated in **A**) and ‘presence-absence’ data (indicated in **B**) as measures of taxonomic representation in the BRS specimen and in the modern (Italian), ethnographic (Hadza and Malawian) and ancient (Ötzi) IM datasets. Weighted (**A**) (as shown Fig. 1C and based on the ‘relative abundance’ of identified IM taxa) (ANOSIM *R* ꞊ 0.6111; *p* ꞊ <0.001) and un-weighted (**B**) Bray-Curtis analysis (based on ‘presence-absence’ of identified IM taxa) exhibit corresponding differences between the ancient and modern IM cohorts (ANOSIM *R* ꞊ 0.8166; *p* ꞊ <0.001).

**Fig. S8.** Bar-plot indicating that the surrounding sedimentary matrix (*i.e.*, samples BRS1 ‘SC1’ and BRS5 ‘SC2’) and the DNA extraction (*n* = 1) and library preparation (*n* = 1) negative controls (‘E-LPCs’) are not noteworthy sources of the twenty-four authenticated ancient microbial taxa identified in the palaeo-faecal specimen (*i.e.*, BRS2, BRS3 and BRS4). Reverse contamination, *i.e.*, from the palaeo-faecal specimen into the surrounding sediment, is most likely responsible for the incidence of low numbers of reads for IM-specific taxa in the surrounding sedimentary matrix (BRS1 and BRS5).

**
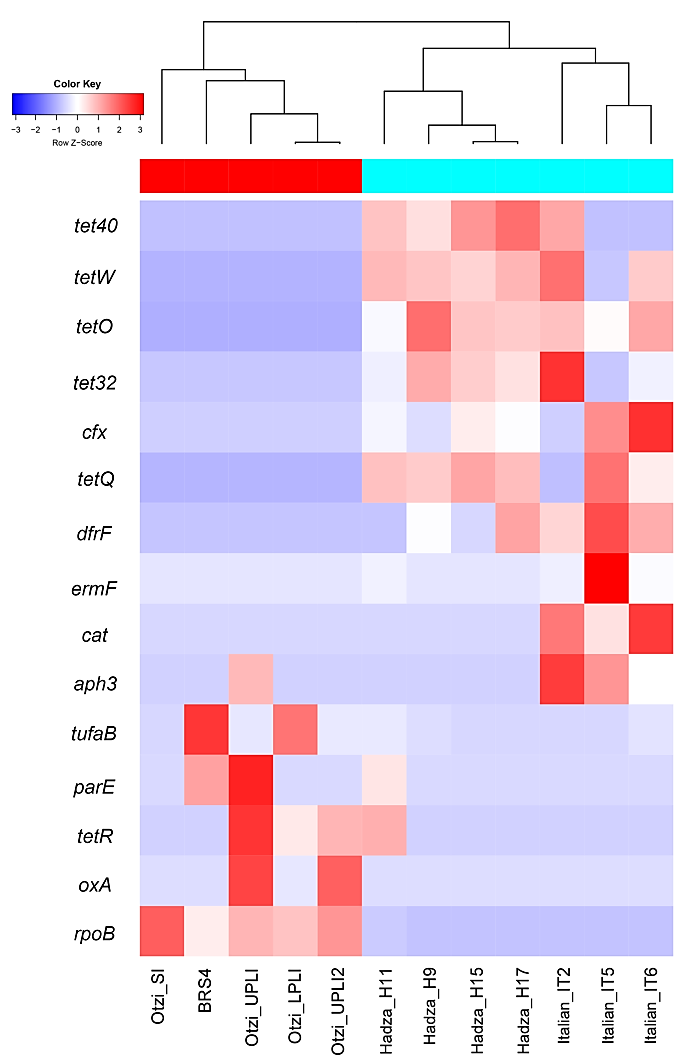
**

**Fig. S9.** Heat-map indicating the presence of fifteen functional ARGs identified in the analysed datasets, four of which occurs in the BRS IM, including the prokaryotic protein synthesis elongation factor Tu (EF-Tu) (*tufA* and *tufB*), flouro-quinolene-resistant DNA topoisomerase (*parE*) and daptomycin-resistant *rpoB*. ARG categories were filtered for occurrence of >5 in at least 20% of samples.

**Supplementary Tables**

**Table S1.** Mapped DNA sequence reads for environmental- and subsistence-related taxa detected in this study. Statistically-significant (*i.e.*, verified ancient) C-T *p*-values are indicated in bold text. Analyses were performed using high-quality filtered read alignments against NCBI reference genomes. DNA damage estimation analyses was performed using PMDtools (‘C-T *p*-values’).

**Table S2.** Information concerning the two direct radiocarbon (^14^C) Accelerator Mass Spectrometry (AMS) dates generated from two sub-samples taken from within the BRS palaeo-faecal specimen.

**Table S3a.** Processing protocol and results for isotope analyses of samples derived from the BRS specimen indicating the relative proportions of C3 and C4 dietary contributions.

**Table S3b.** Results for isotope analyses (Merck standard) of samples derived from the BRS specimen indicating the relative proportions of C3 and C4 dietary contributions.

**Table S3c.** Results for isotope analyses (DL-Valine standard) of samples derived from the BRS specimen indicating the relative proportions of C3 and C4 dietary contributions.

**Table S4.** Assignment of abundance of bacterial taxonomic categories to the BRS and ancient (Ötzi) and modern (Italian, Hadza and Malawian) comparative samples based on *p*-value (*p* ꞊ <0.05) designation. Group significance assignments were obtained via Qiime v1.9.1.

**Table S5.** Information concerning DNA sequence read-length distribution for taxa identified in this study. Read-length distributions were calculated from BWA alignments and BAM files.

**Table S6.** Relative abundance of eighteen significant KEGG pathways detected in BRS and the ancient (Ötzi) and modern (Italian, Hadza and Malawian) comparative IM cohorts**.**

**Table S7.** Enrichment and depletion of KO metabolic gene categories in the BRS and ancient (Ötzi) and modern (Italian, Hadza and Malawian) comparative IM sample cohorts based on *p*-value (*p* ꞊ <0.05) designation. Group significance analyses were performed using Qiime v1.9.1.

**Table S8.** Enrichment and depletion of KO metabolic gene categories in the ancient (BRS and Ötzi) and modern (Italian, Hadza and Malawian) comparative IM sample cohorts based on false discovery rate (FDR) corrected *p*-values (*q* ꞊ <0.05).

**Table S9.** Enrichment and depletion of KO metabolic gene categories in the ancient and modern comparative IM sample cohort as calculated for the twenty-four authenticated ancient IM taxa.

**Table S10.** Comparison of relative abundance of antibiotic resistance genes in the BRS and the ancient (Ötzi) and modern (Italian, Hadza and Malawian) comparative IM cohorts.

**Table S11.** Raw and filtered high-quality sequence read counts as related to the BRS and the ancient (Ötzi) and modern (Italian, Hadza and Malawian) comparative IM datasets.

**Table S12.** Information concerning the comparative NCBI genomes used during this study.
